# Prediction of necrotizing enterocolitis in very low birth weight infants by superior mesenteric artery ultrasound of postnatal day 1: A nested prospective study

**DOI:** 10.3389/fped.2022.1102238

**Published:** 2023-01-16

**Authors:** Guang Yue, Jun Wang, Sheng Yang, Ying Deng, Yang Wen, Wen Jia, Huiling Cao, Rong Ju, Yuan Shi

**Affiliations:** ^1^ Chongqing Key Laboratory of Child Infection and Immunity, Children’s Hospital, Chongqing Medical University, Chongqing, China; ^2^ Neonatal Department, Children’s Hospital of Chongqing Medical University, Chongqing, China; ^3^ National Clinical Research Center for Child Health and Disorders, Chongqing, China; ^4^ Ministry of Education Key Laboratory of Child Development and Disorders, Children’s Hospital of Chongqing Medical University, Chongqing, China; ^5^ Neonatal Department, Chengdu Women’s and Children’s Central Hospital, Chongqing, China; ^6^ Ultrasonography Department, Chengdu Women’s and Children’s Central Hospital, Chongqing, China

**Keywords:** doppler ultrasound, necrotizing enterocolitis, very low birth weight infant, prediction, superior mesenteric artery

## Abstract

**Background:**

Necrotizing enterocolitis (NEC) is a devastating intestinal complication that occurs mainly in very-low-birth-weight infants (VLBWI). The study's aim was to investigate the possibility of early prediction of NEC on postnatal day 1 based on superior mesenteric artery (SMA) doppler ultrasonograpy.

**Methods:**

A prospective, observational, nested case control study (ChiCTR1900026197) was conducted to enroll VLBWIs (birth weight <1,500 grams) between October 2019 and September 2021. Doppler ultrasound measurement was done during the first 12 h of life and before first feeding. Infants developing NEC (stage II or III) subsequently were included in NEC group and infants spare of NEC were included in control group.

**Results:**

370 VLBWIs were enrolled (30 NEC cases). Among the ultrasound parameters, S/D was significantly higher in the NEC group (OR: 2.081, 95% CI: 1.411–3.069, *P *= 0.000). The area under the receiver operating curve (AUROC) following the Logistic regression was 0.704 (95% CI: 0.566–0.842, *P *= 0.001). The sensitivity of S/D for predicting NEC was 52.2% and the specificity was 92.7%. The critical value of S/D was 6.944 and Youden index was 0.449. Preplanned subgroup analysis confirmed that NEC infants of different stages were characterized by different SMA bloodstream. Small for gestational age (SGA) might be a confounding factor affecting intestinal bloodflow. And infants with delayed initiation or slow advancement of feeding exhibited characteristic intestinal perfusion.

**Conclusions:**

In VLBWI, early SMA ultrasound shows the potential to predict NEC. It is reasonable to speculate that SMA bloodstream is related to intestinal structural and functional integrity.

## Introduction

Annually, about 15 million preterm infants (gestational age <37 weeks) are born globally (1), with an incidence of approximately 11%. Immaturity has been an important cause of neonatal mortality and even mortality of children under 5 years of age (2, 3). As an abrupt onset intestinal complication, neonatal necrotizing enterocolitis (NEC) is a multi-factor-related disease that mainly occurs in premature-birth infants. In very-low-birth-weight infants (VLBWI, birth weight <1,500 grams), the morbidity of NEC is 5.1%–9% (4–6), and surgical treatment can account for more than 50% of them (8,935/17,159) (7). The mortality rate of NEC is as high as 30%–50% (7–9), and complications such as intestinal stenosis and intestinal insufficiency may occur even if they survive. Moreover, the incidence of NEC-related long-term adverse neurological prognosis also reached 24%–59.3% (9–12). Generally, neonatology has been developing rapidly in recent decades, but it seems that efforts to control the onset of NEC have not been successful, let alone long-term outcomes.

Besides, there are also studies dedicated to predicting NEC and helping clinical work by identifying high-risk groups. The goal of these studies is to improve the prognosis of preterm infants, and ultrasound is one of the explorations. Superior mesenteric artery (SMA), a major branch of the descending aorta, is the main source of blood supply to the intestine (13). Our previous study tentatively confirmed that preterm infants with NEC have different SMA doppler ultrasound characteristics on first postnatal day, which indicates the potential of this technology to predict NEC (14). Next, we will focus on VLBWIs. This study intended to verify the predictive ability of subsequent NEC onset by SMA doppler ultrasound on the first day of life, and to evaluate whether the prediction model could be constructed through bloodstream indicators.

## Materials and methods

### Study design

This is a prospective, observational, nested case-control study (Chinese Clinical Trial Registry: ChiCTR1900026197). It was approved by hospital Ethics Committee [2019 (10)]. Enrollment took place between October 2019 and September 2021. Guardians, staffs of neonatal and radiological departments were blinded to the measurements of SMA doppler ultrasound.

Inclusion criteria: (1) These infants were born in our hospital, and admitted into neonatal department quickly after birth to receive complete support and treatment; (2) Birth weight should be less than 1,500 grams; (3) Written informed consents were obtained from guardians; (4) Doppler ultrasonography should be done within 12 h of birth, when the respiration and circulation were stable. In this study, cardiopulmonary stability refers to preductal SaO_2_ 90%–95%, mean blood pressure ≥30 mmHg, arterial pH > 7.25, PaCO_2_ 35–45 mmHg, PaO_2_ 50–80 mmHg.

Exclusion criteria: (1) Refused to provide consent; (2) Participated in other studies; (3) Not admitted to neonatal department shortly after birth or just received limited supportive care at the family’s request; (4) Unable to achieve cardiopulmonary stability within 12 h; (5) Feeding couldn’t be initiated during hospitalization; (6) Major or lethal congenital anomalies.

Preterm infants management protocol of the neonatal intensive care unit (NICU) recommended that first feeding should be initiated no later than 12–24 h of life, and the ultrasound should be completed before it. Infants who developed NEC (stage II–III) during hospitalization were included in NEC group, and those without NEC were included in the control group.

### Doppler ultrasound

SMA bloodstream doppler ultrasound were done by three experienced ultrasonologists, and interobserver agreement was tested prior to the study. Bloodstream were measured by CX50 ultrasound machine (Philips Healthcare, Bothell, WA) with L12–3 linear probe (3–12 MHz).

The probe was placed vertically and softly below the xiphoid process of the lying infant during the whole examination. The sample volume of doppler ultrasound was set near the origin of SMA with acceptable angle between soundwave and bloodstream (<30°). For measurement and calculation, continuous recording of at least 5 cardiac cycles was necessary. The average of three tests was the final result of the ultrasound examination. Direct indicators include: peak systolic velocity (PSV), end-diastolic velocity (EDV), time-averaged mean velocity (TAMV). Calculated parameters include: differential velocity (DV, DV = PSV–EDV), SD index (SD = PSV/EDV), pulsatility index [PI, PI = (PSV–EDV)/TAMV], and resistance index [RI, RI = (PSV–EDV)/PSV].

### Clinical management

Breast milk is the default choice, and the formula is also an alternative when breast milk is unavailable.

According to modified Bell`s criteria (15, 16), NEC stage I (suspected) refers to nonspecific signs (heart rate <100 bpm, apnea, poor response, body temperature fluctuation) and intestinal abnormalities (feeding intolerance, bloody stool). The manifestations of stage II (definite) refers to abdominal muscular tension, disappearance of bowel sounds. Infants with x-ray confirmed pneumatosis intestinalis were diagnosed NEC IIA, and NEC IIB was featured by both x-ray confirmed portal venous gas and pneumatosis intestinalis. Infants progress to NEC IIIA could show obvious systemic complications (shock, sepsis, acidosis), severe abdominal conditions such as ascites. Infants with intestinal perforation are diagnosed NEC IIIB. To reduce risk of bias, the diagnosis of NEC should be made jointly by two neonatologists and one radiologist.

### Study population

G*Power (Version 3.1.9.6) was used for sample size calculation. Historical data at our hospital showed an 8% incidence of NEC in VLBWIs. According to our previous study, it was assumed that the DV was 45 cm/s (standard deviation ±18 cm/s) in the NEC group and 34 cm/s (standard deviation ±13 cm/s) in the non-NEC group. With 5% significance, 90% power and 20% loss-to-follow, 328 VLBWIs (28 NEC infants and 300 Non-NEC infants) were required.

### Data management

The endpoint of the study was a diagnosis of NEC (stage II or III) during hospitalization. Information of obstetric conditions, perinatal history, illnesses and treatments, ultrasound measurements, nutritions was collected. Given the excellent follow-up rate, the handling of missing data was done by removal of incomplete cases.

Continuous variables with non normal distribution were expressed as medians and quartiles, and compared by using the Mann–Whitney *U* test. Categorical variables (numbers and percentages) were tested by *χ*^2^ or Fisher's exact test as appropriate. Factors that had significant differences in the univariate test and passed the collinearity test were included in the logistic regression analysis. A receiver operating characteristic curve could be drawn based on the logistic regression analysis. All tests were two-sided and *P *< 0.05 were considered to be significantly different. All data analyses were performed using SPSS version 22.0 software (IBM Corporation, Armonk, NY).

Preplanned subgroup analyses include: (1) NEC stage II vs. stage III, to reveal if severity of NEC is related with intestinal bloodflow; (2) The control group was divided into two subgroups (1000–1,499 grams vs. <1,000 grams) to verify the relationship between birth weight and SMA bloodstream; (3) Control group was divided into ≥32 gestational weeks and <32 gestational weeks subgroups to test the relation between ultrasound and gestational age; (4) Infants diagnosed small for gestational age (SGA) (birth weight below the 10% of the same gestational week) were selected to evaluate the effect of fetal growth restriction on intestinal bloodflow.

As the major supply of blood perfusion in the gut, SMA is of great importance for the structural and functional integrity of the intestine. Thus we conducted further exploratory analyses to assess the association of intestinal perfusion stability with feeding of VLBWI.

## Results

**Figure 1:**
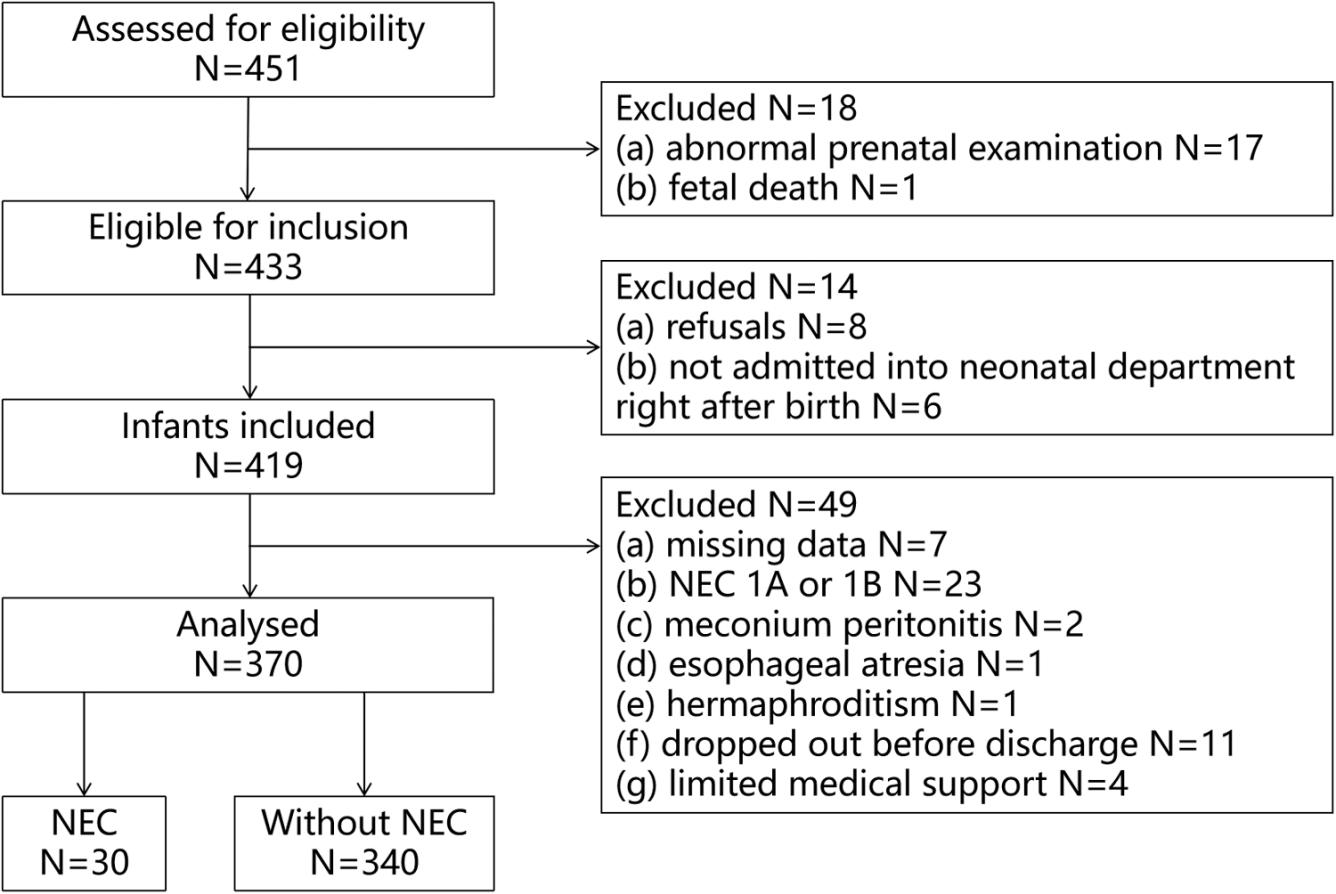
Flowchart of the study.

A total of 451 VLBWIs were screened for eligibility during the investigation, 433 infants met the inclusion criteria (17 abnormal pregnancy, 1 stillbirth). 419 VLBWIs were included (8 refused to participate, 6 not admitted quickly after birth). Finally there were 370 VLBWIs entered the data analysis (Figure 1).

30 VLBWIs diagnosed with NEC stage II or III were included in the NEC group, while 340 VLBWIs without NEC served as control group. Details of NEC infants were listed in the Supplementary Material. Demographics, obstetric conditions, perinatal events, postnatal comorbidities and treatments were compared between two groups (Table 1). The breastfeeding rate in the NEC group was significantly lower than that in the control group [18 (60.00%) vs. 277 (81.47%), *P *= 0.009], and the NEC group infants needed longer days to reach 120 ml/kg.d of feeding (days) [16 (12, 21) vs. 14 (11, 17), *P *= 0.026]. According to other endpoints, the NEC group had significantly higher rates of late-onset sepsis [8 (26.67%) vs. 12 (3.53%), *P *= 0.000], longer hospital stays (days) [61 (42, 66) vs. 41 (33, 50), *P *= 0.000], and significantly higher in-hospital mortality than the control group [4 (13.33%) vs. 6 (1.76%), *P *= 0.005].

**Table 1:** Demographic, obstetric, and comorbidity.

Characteristic	NEC (*n* = 30)	Control (*n* = 340)	*P*
Demographics			
Gestational age, days (median, IQR)	29 ^+ 3^ (27 ^+ 6^–31 ^+ 1^)	30 ^+ 1^ (28 ^+ 6^–31 ^+ 2^)	0.155
Birth weight, grams (median, IQR)	1210.0 (1035.0–1327.5)	1280.0 (1131.3–1400.0)	0.086
Small for gestational age, *n* (%)	8 (26.67)	73 (21.47)	0.495
Maternal Factors			
In vitro fertilization, *n* (%)	7 (23.33)	88 (25.88)	1.000
Chorioamnionitis, *n* (%)	1 (3.33)	7 (2.06)	0.495
Hypertension, *n* (%)	1 (3.33)	35 (10.29)	0.338
Diabetes mellitus, *n* (%)	1 (3.33)	36 (10.59)	0.339
Cholestasis, *n* (%)	1 (3.33)	18 (5.29)	1.000
Perinatal Factors			
Dexamethasone, *n* (%)	8 (26.67)	139 (40.88)	0.172
Natural delivery, *n* (%)	16 (53.33)	156 (45.88)	0.451
1 min Apgar score, points (median, IQR)	8 (7–8)	8 (7–8)	0.919
5 min Apgar score, points (median, IQR)	9 (8–9)	9 (8–9)	0.976
Ventilation, *n* (%)	8 (26.67)	82 (24.12)	0.824
Intubation, *n* (%)	5 (16.67)	69 (20.29)	0.813
Chest compression, *n* (%)	1 (3.33)	15 (4.41)	1.000
Adrenaline, *n* (%)	1 (3.33)	9 (2.65)	0.575
Comorbidities			
Respiratory distress syndrome, *n* (%)	24 (80.00)	234 (68.82)	0.299
Bronchopulmonary dysplasia, *n* (%)	7 (23.33)	55 (16.18)	0.311
Early onset sepsis, *n* (%)	9 (30.00)	66 (19.41)	0.163
Late onset sepsis, *n* (%)	8 (26.67)	12 (3.53)	<0.001
Intraventricular hemorrhage, *n* (%)	6 (20.00)	74 (21.76%)	1.000
Hemodynamically significant PDA, *n* (%)	5 (16.67)	35 (10.29)	0.350
Treatments			
Noninvasive ventilation before 7 days, *n* (%)	12 (40.00)	112 (32.94)	0.427
Mechanical ventilation before 7 days, *n* (%)	15 (50.00)	141 (41.47)	0.441
Medicine for PDA, *n* (%)	2 (6.67)	19 (5.59)	0.683
PDA ligation, *n* (%)	0 (0.00)	4 (1.18)	1.000
Blood transfusion before NEC onset, *n* (%)	4 (13.33)	38 (11.18)	0.955
Feeding			
Breast milk, *n* (%)	18 (60.00)	277 (81.47)	0.009
Fasting> first 24 h, *n* (%)	9 (30.00)	53 (15.59)	0.069
120 ml/kg.d, days (median, IQR)	16 (12–21)	14 (11–17)	0.026
Length of hospital stay, days (median, IQR)	61 (42–66)	41 (33–50)	<0.001
Hospital mortality, *n* (%)	4 (13.33)	6 (1.76)	0.005

NEC, necrotizing enterocolitis; PDA, patent ductus arteriosus.

Univariate analysis of SMA doppler ultrasound between two groups detected significant differences in multiple parameters (Table 2). The risk of NEC was significantly higher as EDV and TAMV decreased. The DV, S/D, PI and RI of the NEC group were significantly higher than those of the control group. There was no significant difference in the proportion of absent SMA blood flow during diastole [4 (13.33%) vs. 23 (6.76%), *χ*^2 ^= 0.921, *P *= 0.337], and there was also no significant difference in reverse flow during diastole [3 (10.00%) vs. 14 (4.12%), *χ*^2 ^= 1.041, *P *= 0.308].

**Table 2:** SMA Doppler ultrasound parameters.

Parameters	NEC (*n* = 30)	Control (*n* = 340)	*Z*	*P*
PSV, cm/s (median, IQR)	46.1 (38.6–54.1)	41.3 (32.4–52.2)	−1.641	0.101
EDV, cm/s (median, IQR)	5.8(2.5–11.0)	9.9 (7.3–12.9)	−3.185	0.001
TAMV, cm/s (median, IQR)	11.0 (6.6–17.5)	16.0 (11.7–20.3)	−2.728	0.006
DV, cm/s (median, IQR)	39.4 (32.5–52.5)	32.3 (24.1–42.3)	−2.612	0.009
S/D (median, IQR)	6.951(3.991–17.087)	4.176(3.326–5.373)	−3.260	0.001
PI (median, IQR)	4.567 (1.868–6.284)	2.026 (1.419–2.784)	−3.553	<0.001
RI (median, IQR)	0.913(0.763–0.973)	0.772(0.711–0.835)	−3.676	<0.001

**Figure 2:**
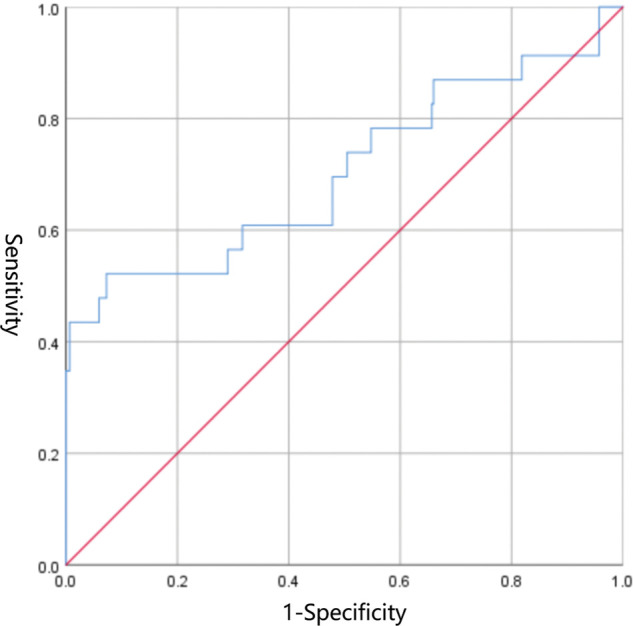
Receiver operating curve of S/D.

EDV was eliminated in the collinearity test of the regression analysis (see Supplementary Material). Subsequent Logistic regression analysis confirmed that S/D was significantly associated with NEC (OR: 2.081, 95% CI: 1.411–3.069, *P *= 0.000) (Table 3). The area under the receiver operating curve (AUROC) following the Logistic regression analysis was 0.704 (95% CI: 0.566–0.842, *P *= 0.001) (Figure 2). The sensitivity of S/D for predicting NEC was 52.2% and the specificity was 92.7%. The critical value of S/D was 6.944 and Youden index was 0.449.

**Table 3:** Logistic regression for the risk of NEC.

							95% CI for Exp (B)	
	B	S.E.	Wald	df	Sig.	Exp (B)	Lower	Upper
Breast milk	0.990	0.575	2.965	1	0.085	2.692	0.872	8.308
120 ml/kg.d, day	0.018	0.033	0.307	1	0.579	1.018	0.955	1.086
TAMV, cm/s	0.086	0.065	1.752	1	0.186	1.089	0.960	1.236
DV, cm/s	−0.065	0.046	2.031	1	0.154	0.937	0.856	1.025
S/D	0.733	0.198	13.687	1	<0.001	2.081	1.411	3.069
PI	−0.014	0.316	0.002	1	0.965	0.986	0.531	1.833
RI	−4.826	5.184	0.867	1	0.352	0.008	0.000	207.471
Constant	−2.709	3.574	0.575	1	0.448	0.067		

Preplanned subgroup analyses were detailed in the Supplementary Material. (1) 30 VLBWIs were diagnosed NEC (stage II or III), the age of onset was 22 (17, 28) days, 23 (76.67%) patients presented with pneumatosis intestinalis on AXR, 17 (56.67%) presented with portal venous gas on AXR, 14 (46.67%) received surgical treatment. In the comparison of ultrasound parameters, NEC patients of stage II presented lower PSV compared with stage III (cm/s) [44.0 (36.2, 48.5) vs. 54.8 (41.2, 63.8) (cm/s), *P *= 0.039], and stage II patients also had lower DV (cm/s) [34.8 (32.5, 40.3) vs. 52.8 (33.2, 60.6), *P *= 0.039]. (2) Among the control group of 340 VLBWIs spared of NEC, there were 35 extremely low birth weight infants (ELBWI, birth weight <1,000 grams), other infants’s birth weight are larger than ELBWIs (1000–1,499 grams). Although significant differences naturally existed between the two subgroups in terms of demographics, diseases, treatments, and nutritions, but there was no significant difference in ultrasound parameters between them. (3) Among the control group of 340 VLBWIs spared of NEC, there were 57 infants with gestational age ≥32 weeks and 283 infants with gestational age <32 weeks. Although there were significant differences in multiple indicators of baseline characteristics between the two subgroups, ultrasound parameters did not show significant differences between them. (4) Among the control group of 340 VLBWIs spared of NEC, there were 73 SGA infants and 267 non-SGA infants, differences existed in a number of demographics between the two subgroups. Furthermore, obvious differences could be detected in ultrasound parameters.

Exploratory analyses were also detailed in the Supplementary Material. (1) Of the 370 VLBWIs, 308 infants started feeding within 24 h, and 62 infants’ feedings were introduced after 24 h. There were significant differences in multiple ultrasound parameters between the two groups. (2) The feeding volume of 86 infants reached 120 ml/kg.d within 10 days of age, while 284 infants did not reach it until 10 days or even later. Significant differences in multiple ultrasound parameters could be seen between the two groups.

During the ultrasound examination, no infant had adverse reactions.

## Discussion

In this prospective, observational, nested case-control study of 370 VLBWIs, we found differences in multiple parameters of SMA ultrasound between 2 groups, and we finally confirmed the relation between S/D and incidence of NEC by Logistic regression analysis. The receiver operating characteristic curve also preliminarily verified the reasonable predictive power of this ultrasound parameter. Subgroup analysis unveiled that SMA bloodstream was related to the severity of NEC.

Murdoch et al. reported that EDV, TAMV, and PI of first day’s SMA ultrasound were related to NEC of preterm infants (17). 104 preterm infants were included in our previous study, DV was significantly associated with NEC (14). Both studies highlighted the importance of hemodynamic stability in terms of early stage SMA ultrasound parameters. This study further demonstrated that in VLBWI, the instability of gut perfusion in the early postnatal days was closely related to the incidence of NEC.

Since NEC seriously affects the prognosis of preterm infants, studies have been devoted to predicting this devastating disease. Alice et al. included 40 extremely preterm infants (gestational age <28 weeks), 11 developed NEC, 29 did not, none of 189 serum biomarkers within 2 days of age could effectively detect the risk (18). Although there have been many studies on biomarkers, none succeeded to exhibit good predictive power (19, 20). Ultrasound is another research direction. Prior studies have suggested the possible relationship between early postnatal ultrasound and the onset of NEC (14, 17). This is only a preliminary study, but the hypothesis has also been confirmed in VLBWIs. The result will undoubtedly encourage more similar studies to further explore the potential of early postnatal SMA ultrasound in predicting NEC.

In neonatal (21, 22) or animal (23, 24) studies, ultrasonography in the early stages of NEC suggests greater intestinal circulatory resistance and hemodynamic instability. Consistent with other studies, our finding extrapolates the relationship between high-resistance intestinal bloodstream and NEC to early postnatal stage. In addition to cardiac function and blood volume, local perfusion is also regulated by some factors (25). Miyake et al. found that mice knocked out of endothelin receptor B (to regulate vasoconstriction) exhibited less severe NEC, which underscores the important role of perfusion (26). It is worth noting the uncertainty of a direct connection between early intestinal perfusion and subsequent onset of NEC. Further exploration is required to reveal the pathophysiological basis for early prediction of NEC by ultrasound.

SMA provides major gut perfusion, and blood supply is crucial for structural and functional integrity, which is also confirmed by our exploratory analysis. Robel et al. found that early feeding volume was affected by intestinal hemodynamic stability after birth, but VLBWIs with poor feeding tolerance were mostly SGA infants in their analysis (27). Jain et al. included 63 VLBWIs, they could not confirm a clear correlation between SMA ultrasound and feeding intolerance, however, they also found significant differences in SMA bloodstream characteristics in SGA infants (28). In our study, multiple parameters of SMA ultrasound of SGA subgroup were very specific. In view of the heterogeneity of these studies in aspects such as subject inclusion, clinical management, ultrasonography, etc, we cannot yet say with certainty that SGA is a key variable affecting SMA blood flow, although SGA itself does represent some abnormalities in fetal growth and development.

VLBWIs are in need of sophisticated and hierarchical management, and SMA ultrasound in early postnatal period provides this possibility. Patel et al. (29) reported that severe anemia was associated with increased risk of NEC rather than red blood cell transfusion itself in a multicenter observational study of 598 VLBWIs. Currently, blood transfusion for VLBWIs was mainly based on age and respiratory support. This study requires us to evaluate the relationship between anemia and NEC especially in high-risk infants, and discuss the benefits and risks of blood transfusion with their families to protect VLBWIs as much as possible. Besides, if the infant's intestinal blood flow is unstable (such as high S/D), umbilical artery catheter (UAC) may need to be removed. Pranav et al. reported that UAC is one of the factors affecting intestinal blood perfusion (30). We can even imagine whether it is possible to adopt individualized feeding plan under ultrasound monitoring for NEC high-risk infants to avoid relative ischemia and reperfusion caused by feeding? (31).

Although our study confirms the relationship between SMA bloodstream and NEC in VLBWIs, obviously there are limitations: (1) NEC stage I (suspected) infants were not included, which helped to distinguish NEC group from control group, but in fact, the in-hospital treatment, nutrition of VLBWIs with suspected NEC are also affected, even not so severe as confirmed NEC. (2) Although no significant difference was observed in the proportion of SGA in 2 groups, subgroup analysis confirmed that SGA was more prone to high-resistance bloodstream, which is consistent with other studies (27, 28). SGA could be an critical factor affecting intestinal blood supply and may also be a confounding factor for intestinal complications, which should be considered in future studies. (3) S/D is of good specificity (92.7%) but suboptimal sensitivity (52.2%), which also results in a imperfect predictive power (AUROC 0.704). It allows us to effectively identify infants with low risk of developing NEC. However, the prediction inevitably has a relatively high false-positive rate. We expect this shortcoming could be overcome after newly developed study and model construction, or even through machine learning. (4) Due to the lack of adequate understanding of the intestinal blood flow in the early postnatal period of VLBWIs, we can only remove the missing data in ultrasonic measurement. (5) Intestinal blood flow changes rapidly in the early postnatal period, so ultrasound should completely reflect both the stability and adaptability. Feeding is one of the key factors affecting gut perfusion (32). Our study reflects the stability of intestinal blood flow before feeding, but it is obviously not an intact mirror, which does not reflect the full picture of intestinal blood flow. Subsequent studies should monitor blood flow before and after feeding.

In conclusion, there is now a potential for early prediction of NEC *via* SMA ultrasonography, which if timely managed, could reduce morbidity and mortality of VLBWIs. We hope that future well-designed studies will further confirm this and ultimately contribute to improving outcomes in preterm infants.

## Data Availability

The original contributions presented in the study are included in the article/**Supplementary Material**, further inquiries can be directed to the corresponding author/s.
