# Supplementary material for: Prediction of necrotizing enterocolitis in very low birth weight infants by superior mesenteric artery ultrasound of postnatal day 1: A nested prospective study
Source: Front Pediatr. 2023 Jan 16;10:1102238. doi: 10.3389/fped.2022.1102238 (PMC9885174; doi:10.3389/fped.2022.1102238)
Supplement: Supplementary file 1 [file Table1.docx]

**Study Protocol**

**Early Prediction of NEC by SMA Ultrasound**

Version 1.2

August, 2019

**Guang Yue**

Neonatal Department, Children`s Hospital of Chongqing Medical University

Chongqing Key Laboratory of Child Infection and Immunity

No. 136, Zhongshan Second Road, Yuzhong District, Chongqing, China

**Yuan Shi**

Neonatal Department, Children`s Hospital of Chongqing Medical University

Chongqing Key Laboratory of Child Infection and Immunity

No. 136, Zhongshan Second Road, Yuzhong District, Chongqing, China

[Team Members 3](#_Toc25728)

[Abbreviation 4](#_Toc18093)

[Study Flowchart 5](#_Toc7709)

[Ultrasound Examination 6](#_Toc18838)

[Ethics 7](#_Toc28348)

[Sample Size 8](#_Toc5617)

[Blinding 9](#_Toc14701)

[Endpoint 10](#_Toc18868)

[Data Management 11](#_Toc12316)

[Data Analysis 12](#_Toc24792)

# Team Members

| **Name** | **Role** | **Contact Information** |
| --- | --- | --- |
| Guang Yue | Principal Investigator | YGmoonlight@139.com |
| Jun Wang | Ethical review submission and statistical analysis | followbeyond@qq.com |
| Sheng Yang | Ultrasound examination | 498259092@qq.com |
| Ying Deng | Ultrasound examination | 87771664@qq.com |
| Yang Wen | Ultrasound examination | 505632737@qq.com |
| Wen Jia | Screening and inclusion, clinical data collection | 512186895@qq.com |
| Huiling Cao | Screening and inclusion, clinical data collection | C16623000406@163.com |
| Rong Ju | Study design, ethics review, and statistical analysis | jurong@uestc.edu.cn |
| Yuan Shi | Study design, statistical analysis, manuscript review | shiyuan@hospital.cqmu.edu.cn |

# Abbreviation

NEC: necrotizing enterocolitis

VLBWI: very low birth weight infant (birth weight <1,500 grams)

SMA: superior mesenteric artery

NICU: neonatal intensive care unit

PSV: peak systolic velocity

EDV: end diastolic velocity

TAMV: time averaged mean velocity

DV: differential velocity

PI: pulsatility index

RI: resistance index

# Study Flowchart

**Screening**

In-hospital delivery

Quick-admission into NICU postnatally

Birth weight < 1,500g

**Inclusion**

Informed consent

Ultrasound within 12 hours after birth

Cardiopulmonary stability*

**Exclusion/Withdrawal**

Refused to participate

Participated in other studies

Cardiopulmonary unstable

Failed to start feeding in NICU

Major or lethal congenital anomalies

**Observation**

Ultrasound within 12 hours after birth and before first feeding

Data collection through discharge

**Grouping**

Group 1: NEC

Group 2: No NEC

**Analysis**

* Cardiopulmonary stability: mean blood pressure ≥30mmHg, SaO_2_ 90-95% of the right upper extremity, pH >7.25 by arterial blood gas analysis, PaCO_2_ 35-45mmHg, PaO_2_ 50-80mmHg.

# Ultrasound Examination

Equipment: CX50 (Philips) with L12-3 linear probe.

Ultrasonologist: Sheng Yang, Ying Deng, Yang Wen

Parameters: PSV, EDV, TAMV, DV, SD index, PI, and RI

DV = PSV – EDV

SD=PSV/EDV

RI=(PSV-EDV)/PSV

PI=(PSV-EDV)/TAMV

Procedure:

1. Coupling agent is warmed.
2. Infant is kept supine position.
3. Location: right below the xiphoid process
4. Measurement: each test should include at least five continuous record cycles, then average the results of three tests.

# Ethics

**Ethical review**

The study was approved by Ethics Committee of Chengdu Women`s and Children`s Central Hospital [2019(10)].

**Informed consent**

The written informed consent from the guardian should be obtained when the infant is admitted into NICU.

**Patient withdrawal**

The participant’s parents are free to withdraw the infant from the study entirely at any time, and this will not have any consequences for the infant’s further treatment.

# Sample Size

We use G*Power (Version 3.1.9.6) to calculate sample size. Incidence of NEC in VLBWIs in our hospital is 8% according to previous result.

Based on our previous study, we assume that DV is 45cm/s (SD ±18cm/s) in NEC group and 34cm/s (SD ±13 cm/s) in control group. It is estimated that 328 VLBWIs (28 NEC infants and 300 Non-NEC infants) will be included with 5% significance, 90% power and 20% loss-to-follow.

# Blinding

In order to avoid infant management based on SMA ultrasound, it is required that ultrasound results should be kept confidential to neonatologists and all care givers. Although we believe that there is no medical evidence for such a treatment plan.

In order to avoid any possible interference with the results of abdominal X-rays, the results of SMA ultrasound should be kept confidential to radiologists.

# Endpoint

The endpoint of this observational study is NEC (stage Ⅱ or Ⅲ) onset during hospitalization.

Diagnosis of NEC will follow modified Bell`s criteria. NEC stage Ⅰ (suspected) refers to nonspecific signs (bradycardia, apnea, temperature instability) and intestinal signs (feeding intolerance, bloody stool); NEC stage Ⅱ (definite) refers to clear evidence of acute intestinal and peritoneal inflammation such as bowel sounds and abdominal muscle tension, abdominal X-ray should reveal positive signs of pneumatosis intestinalis (NEC ⅡA) and portal venous gas (NEC ⅡB); while NEC ⅢA refers to serious condition with obvious systemic signs (shock, sepsis, acidosis) and peritonitis, such as ascites or even intestinal perforation (stage ⅢB).

Diagnosis should be made by 2 neonatologists and an experienced radiologist to control bias.

# Data Management

Infants will be recruited over a period of 24 months. Approximately another 2 months will be needed to collect hospital data on all infants enrolled. All data on each infant will be documented on an electronic sheet.

Data-safety monitoring board will be responsible for supervising the whole process from infant enrollment to statistical analysis.

All data with identifiers will be stored on firewall-protected servers.

# Data Analysis

The primary endpoint is the onset of NEC during hospitalization. Our hypothesis is whether the measurements of SMA ultrasound are significantly different between NEC group and control group. Logistic regression will be utilized to test the variables obtained through univariate analysis.

Prior studies offer no basis for an assumed interaction between subgroups defined by birth weight, gestational age, severity of NEC. Therefore, we expect to list all results subgroups for descriptive purposes and to explore in secondary analysis, hoping to establish consistency and/or generating hypotheses for future studies.

We will also collect the feeding situation of infants during hospitalization, and exploratory analysis will evaluate the correlation between early postnatal intestinal blood flow and feeding.
