# Supplementary material for: Prediction of necrotizing enterocolitis in very low birth weight infants by superior mesenteric artery ultrasound of postnatal day 1: A nested prospective study
Source: Front Pediatr. 2023 Jan 16;10:1102238. doi: 10.3389/fped.2022.1102238 (PMC9885174; doi:10.3389/fped.2022.1102238)
Supplement: Supplementary file 2 [file Datasheet1.docx]

[Data Analysis on the Main Outcome 2](#_Toc18323)

[Collinearity test before Logistic Regression 2](#_Toc4099)

[Subgroup Analysis 3](#_Toc28908)

[NEC Stage Ⅱ vs. Stage Ⅲ 3](#_Toc12600)

[VLBWI (1000-1499g) vs. ELBWI (＜1000g) 5](#_Toc21042)

[≥32 weeks vs. ＜32 weeks 7](#_Toc26841)

[SGA vs. Non SGA 9](#_Toc6803)

[Exploratory Analysis 11](#_Toc15626)

[Fasting ≤24 hours vs. ＞24 hours 11](#_Toc9382)

[Feeding 120ml/kg.d ≤10 days vs. ＞10 days 12](#_Toc24317)

# Data Analysis on the Main Outcome

## Collinearity test before Logistic Regression

Table 2 Collinearity test

|  | Tolerance | VIF |
| --- | --- | --- |
| Breast milk | 0.970 | 1.030 |
| 120ml/kg.d, days | 0.952 | 1.050 |
| EDV, cm/s | 0.089 | 11.251 |
| TAMV, cm/s | 0.133 | 7.542 |
| DV, cm/s | 0.111 | 8.991 |
| S/D | 0.323 | 3.100 |
| PI | 0.376 | 2.662 |
| RI | 0.107 | 9.327 |

Tolerance<0.05 or VIF>10, indicating that the variable has strong collinearity and cannot be included in logistic regression analysis.

# Subgroup Analysis

## NEC Stage Ⅱ vs. Stage Ⅲ

Table 3 Characteristics of NEC infants

| Characteristic | NEC stage Ⅱ (n=18) | NEC stage Ⅲ (n=12) | *P* |
| --- | --- | --- | --- |
| Demographics |  |  |  |
| Gestational age, days (median, IQR) | 30^+4^ (27^+4^-31) | 29^+1^ (28-31^+2^) | 0.787 |
| Birth weight, grams (median, IQR) | 1205.0 (982.5-1413.8) | 1210.0 (1100.0-1295.0) | 0.851 |
| Small for gestational age, n(%) | 5 (27.78) | 3 (25.00) | 1.000 |
| Maternal Factors |  |  |  |
| In vitro fertilization, n(%) | 4 (22.22) | 3 (25.00) | 1.000 |
| Chorioamnionitis, n(%) | 0 (0.00) | 1 (8.33) | 0.400 |
| Hypertension, n(%) | 1 (5.56) | 0 (0.00) | 1.000 |
| Diabetes mellitus, n(%) | 0 (0.00) | 1 (8.33) | 0.400 |
| Cholestasis, n(%) | 1 (5.56) | 0 (0.00) | 1.000 |
| Perinatal Factors |  |  |  |
| Dexamethasone, n(%) | 2 (11.11) | 6 (50.00) | 0.034 |
| Natural delivery, n(%) | 8 (44.44) | 8 (66.67) | 0.284 |
| 1min Apgar score, points (median, IQR) | 8 (6-8) | 8 (7-8) | 0.787 |
| 5min Apgar score, points (median, IQR) | 9 (8-9) | 9 (8-9) | 0.755 |
| Ventilation, n(%) | 5 (27.78) | 3 (25.00) | 1.000 |
| Intubation, n(%) | 3 (16.67) | 2 (16.67) | 1.000 |
| Chest compression, n(%) | 1 (5.56) | 0 (0.00) | 1.000 |
| Adrenaline, n(%) | 1 (5.56) | 0 (0.00) | 1.000 |
| Comorbidities |  |  |  |
| Respiratory distress syndrome, n(%) | 14 (77.78) | 10 (83.33) | 1.000 |
| Bronchopulmonary dysplasia, n(%) | 6 (33.33) | 1 (8.33) | 0.193 |
| Early onset sepsis, n(%) | 6 (33.33) | 3 (25.00) | 0.704 |
| Late onset sepsis, n(%) | 3 (16.67) | 5 (41.67) | 0.210 |
| Intraventricular hemorrhage, n(%) | 3 (16.67) | 3 (25.00) | 0.660 |
| Hemodynamically significant PDA, n(%) | 0 (0.00) | 5 (41.67) | 0.006 |
| Treatments |  |  |  |
| Noninvasive ventilation before 7 days, n(%) | 7 (38.89) | 5 (41.67) | 1.000 |
| Mechanical ventilation before 7 days, n(%) | 9 (50.00) | 6 (50.00) | 1.000 |
| Medicine for PDA, n(%) | 0 (0.00) | 2 (16.67) | 0.152 |
| PDA ligation, n(%) | 0 (0.00) | 0 (0.00) | / |
| Feeding |  |  |  |
| Breast milk, n(%) | 12 (66.67) | 6 (50.00) | 0.458 |
| Fasting＞first 24 hours, n(%) | 5 (27.78) | 4 (33.33) | 1.000 |
| 120ml/kg.d, days (median, IQR) | 15 (12-21) | 19 (14-23) | 0.415 |
| Length of hospital stay, days (median, IQR) | 46.5 (41.8-64.5) | 63.5 (43.5-70.0) | 0.305 |
| Hospital mortality, n(%) | 1 (5.56) | 3 (25.00) | 0.274 |

NEC, necrotizing enterocolitis; PDA, patent ductus arteriosus.

Table 4 Clinical Course of NEC

| Characteristic | NEC infants (n=30) | NEC stage Ⅱ (n=18) | NEC stage Ⅲ (n=12) | *P* |
| --- | --- | --- | --- | --- |
| Date of onset, days (median, IQR) | 22 (17.0-28.0) | 21.5 (16.8-34.0) | 21.5 (16.8-26.0) | 0.626 |
| Pneumatosis intestinalis, n(%) | 23 (76.67) | 14 (77.78) | 9 (75.00) | 1.000 |
| Portal venous gas, n(%) | 17 (56.67) | 8 (44.44) | 9 (75.00) | 0.141 |
| Surgical treatment, n(%) | 14 (46.67) | 4 (22.22) | 10 (83.33) | 0.002 |

Table 5 Comparison of SMA Doppler Ultrasound

| Parameters | NEC stage Ⅱ (n=18) | NEC stage Ⅲ (n=12) | *Z* | *P* |
| --- | --- | --- | --- | --- |
| PSV, cm/s (median, IQR) | 44.0 (36.2-48.5) | 54.8 (41.2-63.8) | -2.054 | 0.039 |
| EDV, cm/s (median, IQR) | 8.7 (2.5-11.6) | 4.6 (-5.5-8.1) | -1.090 | 0.281 |
| No flow during diastole, n(%) | 3 (16.67) | 1 (8.33) | / | 0.632 |
| Reverse flow during diastole, n(%) | 0 (0.00) | 3 (25.00) | / | 0.054 |
| TAMV, cm/s (median, IQR) | 12.4 (5.5-18.5) | 11.0 (6.9-16.5) | -0.169 | 0.884 |
| DV, cm/s (median, IQR) | 34.8 (32.5-40.3) | 52.8 (33.2-60.6) | -2.074 | 0.039 |
| S/D (median, IQR) | 4.250 (3.627-18.767) | 8.890 (5.620-15.290) | -0.452 | 0.681 |
| PI (median, IQR) | 2.433 (1.629-6.442) | 5.310 (2.515-5.564) | -0.719 | 0.492 |
| RI (median, IQR) | 0.851 (0.743-0.955) | 0.930 (0.859-1.082) | -1.250 | 0.215 |

## VLBWI (1000-1499g) vs. ELBWI (＜1000g)

Table 6 Demographics

| Characteristic | VLBWI (n=305) | ELBWI (n=35) | *P* |
| --- | --- | --- | --- |
| Demographics |  |  |  |
| Gestational age, days (median, IQR) | 30^+2^ (29^+1^-31^+3^) | 27^+6^ (26^+3^-29^+6^) | <0.001 |
| Birth weight, grams (median, IQR) | 1130.0 (1160.0-1420.0) | 880 (770.0-950.0) | <0.001 |
| Small for gestational age, n(%) | 59 (19.34) | 14 (40.00) | 0.005 |
| Maternal Factors |  |  |  |
| In vitro fertilization, n(%) | 74 (24.26) | 14 (40.00) | 0.044 |
| Chorioamnionitis, n(%) | 7 (2.30) | 0 (0.00) | 1.000 |
| Hypertension, n(%) | 31 (10.16) | 4 (11.43) | 1.000 |
| Diabetes mellitus, n(%) | 35 (11.48) | 1 (2.86) | 0.201 |
| Cholestasis, n(%) | 17 (5.57) | 1 (2.86) | 0.778 |
| Perinatal Factors |  |  |  |
| Dexamethasone, n(%) | 128 (41.97) | 11 (31.43) | 0.230 |
| Natural delivery, n(%) | 135 (44.26) | 21 (60.00) | 0.077 |
| 1min Apgar score, points (median, IQR) | 8 (7-9) | 7 (6-8) | 0.006 |
| 5min Apgar score, points (median, IQR) | 9 (8-9) | 8 (8-9) | <0.001 |
| Ventilation, n(%) | 69 (22.62) | 13 (37.14) | 0.057 |
| Intubation, n(%) | 60 (19.67) | 9 (25.71) | 0.400 |
| Chest compression, n(%) | 14 (4.59) | 1 (2.86) | 0.969 |
| Adrenaline, n(%) | 9 (2.95) | 0 (0.00) | 0.606 |
| Comorbidities |  |  |  |
| Respiratory distress syndrome, n(%) | 202 (66.23) | 32 (91.43) | 0.002 |
| Bronchopulmonary dysplasia, n(%) | 39 (12.79) | 16 (45.71) | <0.001 |
| Early onset sepsis, n(%) | 53 (17.38) | 13 (37.14) | 0.005 |
| Late onset sepsis, n(%) | 10 (3.28) | 2 (5.71) | 0.799 |
| Intraventricular hemorrhage, n(%) | 60 (19.67) | 14 (40.00) | 0.006 |
| Hemodynamically significant PDA, n(%) | 31 (10.16) | 4 (11.43) | 1.000 |
| Treatments |  |  |  |
| Noninvasive ventilation before 7 days, n(%) | 105 (34.43) | 7 (20.00) | 0.085 |
| Mechanical ventilation before 7 days, n(%) | 116 (38.03) | 25 (71.43) | <0.001 |
| Medicine for PDA, n(%) | 16 (5.25) | 3 (8.57) | 0.672 |
| PDA ligation, n(%) | 1 (0.33) | 3 (8.57) | 0.004 |
| Feeding |  |  |  |
| Breast milk, n(%) | 250 (81.97) | 27 (77.14) | 0.487 |
| Fasting＞first 24 hours, n(%) | 45 (14.75) | 8 (22.86) | 0.211 |
| 120ml/kg.d, days (median, IQR) | 13 (10-17) | 17 (14-26) | <0.001 |
| Length of hospital stay, days (median, IQR) | 40 (32-49) | 66 (59-94) | 0.006 |
| Hospital mortality, n(%) | 6 (1.97) | 0 (0.00) | 1.000 |

VLBWI, very low birth weight infant; ELBWI, extremely low birth weight infant; PDA, patent ductus arteriosus.

Table 7 Comparison of SMA Doppler Ultrasound

| Parameters | VLBWI (n=305) | ELBWI (n=35) | *Z* | *P* |
| --- | --- | --- | --- | --- |
| PSV, cm/s (median, IQR) | 41.2 (32.1-52.6) | 42.3 (36.5-51.5) | -0.741 | 0.459 |
| EDV, cm/s (median, IQR) | 9.9 (7.2-13.0) | 9.7 (7.5-11.8) | -0.641 | 0.522 |
| No flow during diastole, n(%) | 19 (6.23) | 4 (11.43) | / | 0.421 |
| Reverse flow during diastole, n(%) | 11 (3.61) | 3 (8.57) | / | 0.342 |
| TAMV, cm/s (median, IQR) | 16.0 (11.7-20.3) | 16.2 (12.0-20.8) | -0.035 | 0.972 |
| DV, cm/s (median, IQR) | 31.8 (23.2-42.0) | 34.2 (27.9-44.2) | -1.511 | 0.131 |
| S/D (median, IQR) | 4.154 (3.314-5.319) | 4.323 (3.605-6.124) | -1.032 | 0.302 |
| PI (median, IQR) | 2.024 (1.414-2.779) | 2.144 (1.459-3.257) | -0.794 | 0.427 |
| RI (median, IQR) | 0.771 (0.706-0.828) | 0.814 (0.725-0.869) | -1.833 | 0.067 |

## ≥32 weeks vs. ＜32 weeks

Table 8 Demographics

| Characteristic | ≥32 weeks (n=57) | ＜32 weeks (n=283) | *P* |
| --- | --- | --- | --- |
| Demographics |  |  |  |
| Gestational age, days (median, IQR) | 32^+5^ (32^+1^-34) | 29^+5^ (28^+4^-30^+5^) | <0.001 |
| Birth weight, grams (median, IQR) | 1380.0 (1190.0-1450.0) | 1260.0 (1100.0-1400.0) | 0.001 |
| Small for gestational age, n(%) | 47 (82.46) | 26 (9.19) | <0.001 |
| Maternal Factors |  |  |  |
| In vitro fertilization, n(%) | 7 (12.28) | 81 (28.62) | 0.010 |
| Chorioamnionitis, n(%) | 0 (0.00) | 7 (2.47) | 0.491 |
| Hypertension, n(%) | 16 (28.07) | 19 (6.71) | <0.001 |
| Diabetes mellitus, n(%) | 4 (7.02) | 32 (11.31) | 0.337 |
| Cholestasis, n(%) | 5 (8.77) | 13 (4.59) | 0.336 |
| Perinatal Factors |  |  |  |
| Dexamethasone, n(%) | 21 (36.84) | 118 (41.70) | 0.496 |
| Natural delivery, n(%) | 5 (8.77) | 151 (53.36) | <0.001 |
| 1min Apgar score, points (median, IQR) | 8 (8-9) | 8 (7-8) | <0.001 |
| 5min Apgar score, points (median, IQR) | 9 (9-9) | 9 (8-9) | <0.001 |
| Ventilation, n(%) | 3 (5.26) | 79 (27.92) | <0.001 |
| Intubation, n(%) | 2 (3.51) | 67 (23.67) | 0.001 |
| Chest compression, n(%) | 0 (0.00) | 15 (5.30) | 0.154 |
| Adrenaline, n(%) | 0 (0.00) | 9 (3.18) | 0.362 |
| Comorbidities |  |  |  |
| Respiratory distress syndrome, n(%) | 14 (24.56) | 220 (77.74) | <0.001 |
| Bronchopulmonary dysplasia, n(%) | 0 (0.00) | 55 (19.43) | <0.001 |
| Early onset sepsis, n(%) | 12 (21.05) | 54 (19.08) | 0.731 |
| Late onset sepsis, n(%) | 2 (3.51) | 10 (3.53) | 1.000 |
| Intraventricular hemorrhage, n(%) | 7 (12.28) | 67 (23.67) | 0.057 |
| Hemodynamically significant PDA, n(%) | 2 (3.51) | 33 (11.66) | 0.065 |
| Treatments |  |  |  |
| Noninvasive ventilation before 7 days, n(%) | 12 (21.05) | 100 (35.34) | 0.036 |
| Mechanical ventilation before 7 days, n(%) | 10 (17.54) | 131 (46.29) | <0.001 |
| Medicine for PDA, n(%) | 0 (0.00) | 19 (6.71) | 0.044 |
| PDA ligation, n(%) | 0 (0.00) | 4 (1.41) | 1.000 |
| Feeding |  |  |  |
| Breast milk, n(%) | 46 (80.70) | 231 (81.63) | 0.870 |
| Fasting＞first 24 hours, n(%) | 11 (19.30) | 42 (14.84) | 0.397 |
| 120ml/kg.d, days (median, IQR) | 12 (10-17) | 14 (11-17) | <0.001 |
| Length of hospital stay, days (median, IQR) | 27.0 (23.5-36.0) | 45.0 (36.0-56.0) | <0.001 |
| Hospital mortality, n(%) | 0 (0.00) | 6 (2.12) | 0.595 |

PDA, patent ductus arteriosus.

Table 9 Comparison of SMA Doppler Ultrasound

| Parameters | ≥32 weeks (n=57) | ＜32 weeks (n=283) | *Z* | *P* |
| --- | --- | --- | --- | --- |
| PSV, cm/s (median, IQR) | 43.1 (33.6-54.5) | 41.2 (32.4-52.0) | -0.745 | 0.456 |
| EDV, cm/s (median, IQR) | 10.6 (7.5-13.4) | 9.7 (7.1-12.8) | -1.300 | 0.194 |
| No flow during diastole, n(%) | 7 (12.28) | 16 (5.65) | / | 0.126 |
| Reverse flow during diastole, n(%) | 1 (1.75) | 13 (4.59) | / | 0.536 |
| TAMV, cm/s (median, IQR) | 14.2 (10.5-23.7) | 16.2 (11.8-20.1) | -0.074 | 0.941 |
| DV, cm/s (median, IQR) | 35.6 (26.6-44.8) | 31.8 (23.4-41.7) | -1.377 | 0.168 |
| S/D (median, IQR) | 4.484 (3.371-5.485) | 4.125 (3.323-5.374) | -0.643 | 0.520 |
| PI (median, IQR) | 2.089 (1.460-3010) | 2.018 (1.419-2.767) | -0.561 | 0.575 |
| RI (median, IQR) | 0.790 (0.719-0.835) | 0.765 (0.706-0.836) | -0.936 | 0.349 |

## SGA vs. Non SGA

Table 10 Demographics

| Characteristic | SGA (n=73) | Non SGA (n=267) | *P* |
| --- | --- | --- | --- |
| Demographics |  |  |  |
| Gestational age, days (median, IQR) | 32^+2^ (31^+2^-33^+2^) | 29^+5^ (28^+4^-30^+5^) | <0.001 |
| Birth weight, grams (median, IQR) | 1200.0 (1050.0-1400.0) | 1300 (1150.0-1410.0) | 0.072 |
| Maternal Factors |  |  |  |
| In vitro fertilization, n(%) | 12 (16.44) | 76 (28.46) | 0.038 |
| Chorioamnionitis, n(%) | 0 (0.00) | 7 (2.62) | 0.351 |
| Hypertension, n(%) | 17 (23.29) | 18 (6.74) | <0.001 |
| Diabetes mellitus, n(%) | 8 (10.96) | 28 (10.49) | 1.000 |
| Cholestasis, n(%) | 7 (9.59) | 11 (4.12) | 0.120 |
| Perinatal Factors |  |  |  |
| Dexamethasone, n(%) | 23 (31.51) | 116 (43.45) | 0.066 |
| Natural delivery, n(%) | 8 (10.96) | 148 (55.43) | <0.001 |
| 1min Apgar score, points (median, IQR) | 8 (7-9) | 8 (7-8) | 0.004 |
| 5min Apgar score, points (median, IQR) | 9 (9-9) | 9 (8-9) | 0.001 |
| Ventilation, n(%) | 8 (10.96) | 74 (27.72) | 0.003 |
| Intubation, n(%) | 5 (6.85) | 64 (23.97) | 0.001 |
| Chest compression, n(%) | 0 (0.00) | 15 (5.62) | 0.080 |
| Adrenaline, n(%) | 0 (0.00) | 9 (3.37) | 0.239 |
| Comorbidities |  |  |  |
| Respiratory distress syndrome, n(%) | 29 (39.73) | 203 (76.03) | <0.001 |
| Bronchopulmonary dysplasia, n(%) | 8 (10.96) | 47 (17.60) | 0.172 |
| Early onset sepsis, n(%) | 14 (19.18) | 52 (19.48) | 0.955 |
| Late onset sepsis, n(%) | 3 (4.11) | 9 (3.37) | 1.000 |
| Intraventricular hemorrhage, n(%) | 11 (15.07) | 63 (23.60) | 0.118 |
| Hemodynamically significant PDA, n(%) | 4 (5.48) | 31 (11.61) | 0.127 |
| Treatments |  |  |  |
| Noninvasive ventilation before 7 days, n(%) | 18 (24.66) | 94 (35.21) | 0.089 |
| Mechanical ventilation before 7 days, n(%) | 19 (26.03) | 122 (45.69) | 0.003 |
| Medicine for PDA, n(%) | 1 (1.37) | 18 (6.74) | 0.138 |
| PDA ligation, n(%) | 0 (0.00) | 4 (1.50) | 0.581 |
| Feeding |  |  |  |
| Breast milk, n(%) | 56 (76.71) | 221 (82.77) | 0.238 |
| Fasting＞first 24 hours, n(%) | 14 (19.18) | 39 (14.61) | 0.340 |
| 120ml/kg.d, days (median, IQR) | 13 (10-18) | 14 (11-17) | 0.765 |
| Length of hospital stay, days (median, IQR) | 36 (26-49) | 43 (35-54) | 0.001 |
| Hospital mortality, n(%) | 0 (0.00) | 6 (2.25) | 0.348 |

SGA, small for gestational age; PDA, patent ductus arteriosus.

Table 11 Comparison of SMA Doppler Ultrasound

| Parameters | SGA (n=73) | Non SGA (n=267) | *Z* | *P* |
| --- | --- | --- | --- | --- |
| PSV, cm/s (median, IQR) | 42.6 (33.7-53.3) | 41.1 (32.4-52.0) | -0.740 | 0.459 |
| EDV, cm/s (median, IQR) | 9.2 (7.3-12.8) | 10.0 (7.3-13.0) | -0.222 | 0.824 |
| No flow during diastole, n(%) | 10 (13.70) | 13 (4.87) | / | 0.016 |
| Reverse flow during diastole, n(%) | 3 (4.11) | 11 (4.12) | / | 1.000 |
| TAMV, cm/s (median, IQR) | 13.7 (10.2-21.7) | 16.2 (12.5-20.3) | -1.065 | 0.287 |
| DV, cm/s (median, IQR) | 35.6 (26.8-44.5) | 31.4 (22.9-41.5) | -1.989 | 0.047 |
| S/D (median, IQR) | 4.469 (3.549-5.731) | 4.124 (3.284-5.319) | -1.370 | 0.171 |
| PI (median, IQR) | 2.327 (1.559-3.172) | 1.980 (1.399-2.702) | -1.902 | 0.057 |
| RI (median, IQR) | 0.795 (0.727-0.850) | 0.765 (0.705-0.827) | -2.239 | 0.025 |

# Exploratory Analysis

## Fasting ≤24 hours vs. ＞24 hours

Table 12 Comparison of Feeding Initiation

| Parameters | Fasting ≤24 hours (n=308) | Fasting >24 hours (n=62) | *Z* | *P* |
| --- | --- | --- | --- | --- |
| PSV, cm/s (median, IQR) | 41.8 (33.1-52.0) | 46.1 (32.3-53.3) | -0.752 | 0.452 |
| EDV, cm/s (median, IQR) | 9.9 (7.2-13.1) | 8.7 (6.3-12.1) | -1.678 | 0.093 |
| No flow during diastole, n(%) | 17 (5.52) | 10 (16.13) | / | 0.008 |
| Reverse flow during diastole, n(%) | 13 (4.22) | 4 (6.45) | / | 0.665 |
| TAMV, cm/s (median, IQR) | 16.2 (11.6-21.4) | 13.5 (7.7-17.5) | -3.036 | 0.002 |
| DV, cm/s (median, IQR) | 31.9 (24.1-42.3) | 36.4 (27.6-45.7) | -1.890 | 0.059 |
| S/D (median, IQR) | 4.155 (3.267-5.401) | 4.591 (3.836-6.221) | -2.149 | 0.032 |
| PI (median, IQR) | 1.937 (1.399-2.727) | 2.781 (1.841-3.755) | -4.357 | <0.001 |
| RI (median, IQR) | 0.769 (0.706-0.836) | 0.807 (0.752-0.843) | -3.230 | 0.001 |

## Feeding 120ml/kg.d ≤10 days vs. ＞10 days

Table 13 Comparison of Feeding Advancement

| Parameters | ≤10 days (n=86) | >10 days (n=284) | *Z* | *P* |
| --- | --- | --- | --- | --- |
| PSV, cm/s (median, IQR) | 43.1 (35.9-53.1) | 41.5 (32.4-52.3) | -1.127 | 0.260 |
| EDV, cm/s (median, IQR) | 10.7 (7.9-14.1) | 9.3 (6.6-12.4) | -2.880 | 0.004 |
| No flow during diastole, n(%) | 5 (5.81) | 22 (7.75) | / | 0.546 |
| Reverse flow during diastole, n(%) | 0 (0.00) | 17 (5.99) | / | 0.042 |
| TAMV, cm/s (median, IQR) | 17.5 (13.1-24.2) | 15.0 (10.4-18.7) | -3.299 | 0.001 |
| DV, cm/s (median, IQR) | 33.0 (25.0-42.0) | 33.4 (24.8-43.3) | -0.150 | 0.881 |
| S/D (median, IQR) | 4.061 (3.100-5.219) | 4.254 (3.405-5.557) | -1.382 | 0.167 |
| PI (median, IQR) | 1.785 (1.306-2.450) | 2.235 (1.537-3.016) | -3.163 | 0.002 |
| RI (median, IQR) | 0.758 (0.684-0.817) | 0.780 (0.719-0.854) | -2.331 | 0.020 |
